# A descriptive analysis of the spatio-temporal distribution of enteric diseases in New Brunswick, Canada

**DOI:** 10.1186/s12889-016-2779-5

**Published:** 2016-03-01

**Authors:** James E. Valcour, Dominique F. Charron, Olaf Berke, Jeff B. Wilson, Tom Edge, David Waltner-Toews

**Affiliations:** Division of Community Health and Humanities, Faculty of Medicine, Memorial University of Newfoundland, St. John’s, A1B 3V6 Newfoundland and Labrador Canada; Department of Population Medicine, Ontario Veterinary College, University of Guelph, Guelph, N1G 2W1 ON Canada; International Development Research Centre, Ottawa, ON Canada; Aquatic Ecosystem Protection Research Branch, National Water Research Institute, Environment Canada, Burlington, ON Canada

**Keywords:** Enteric disease, *Salmonella*, *Escherichia coli*, *Campylobacter*, *Giardia*, *Shigella*, Spatial scan statistics, Spatio-temporal epidemiology, GIS, Zoonotic foodborne disease

## Abstract

**Background:**

Enteric diseases affect thousands of Canadians annually and several large outbreaks have occurred due to infection with enteric pathogens. The objectives of this study were to describe the spatial and temporal distributions of reportable *Campylobacter*, *Escherichia coli*, *Giardia*, *Salmonella* and *Shigella* from 1994 to 2002 in New Brunswick, Canada. By examining the spatial and temporal distributions of disease incidence, hypotheses as to potential disease risk factors were formulated.

**Methods:**

Time series plots of monthly disease incidence were examined for seasonal and secular trends. Seasonality of disease incidence was evaluated using the temporal scan statistic and seasonal–trend loess (STL) decomposition methods. Secular trends were evaluated using negative binomial regression modeling. The spatial distribution of disease incidence was examined using maps of empirical Bayes smoothed estimates of disease incidence. Spatial clustering was examined by multiple methods, which included Moran’s *I* and the spatial scan statistic.

**Results:**

The peak incidence of *Giardia* infections occurred in the spring months. *Salmonella* incidence exhibited two peaks, one small peak in the spring and a main peak in the summer. *Campylobacter* and *Escherichia coli* O157 disease incidence peaked in the summer months. Moran’s I indicated that there was significant positive spatial autocorrelation for the incidence of *Campylobacter*, *Giardia* and *Salmonella*. The spatial scan statistic identified clusters of high disease incidence in the northern areas of the province for *Campylobacter*, *Giardia* and *Salmonella* infections. The incidence of *Escherichia coli* infections clustered in the south-east and north-east areas of the province, based on the spatial scan statistic results. *Shigella* infections had the lowest incidence rate and no discernable spatial or temporal patterns were observed.

**Conclusions:**

By using several different spatial and temporal methods a robust picture of the spatial and temporal distributions of enteric disease in New Brunswick was produced. Disease incidence for several reportable enteric pathogens displayed significant geographic clustering indicating that a spatially distributed risk factor may be contributing to disease incidence. Temporal analysis indicated peaks in disease incidence, including previously un-reported peaks.

## Background

Every year, thousands of individuals in Canada become ill due to infection with enteric pathogens. According to the Public Health Agency of Canada, infection with *Campylobacter* spp., *Salmonella* and *Giardia* are the most common causes of infectious enteric disease [1] in Canada. Several large, notable outbreaks have been associated with these pathogens [2–4]. It is estimated that enteric illness affects approximately one in ten Canadians once under-reporting of disease incidence is accounted for [5].

Gastroenteritis can occur due to infection with viral, bacterial or parasitic pathogens. Infection with most enteric pathogens typically results in self-limiting disease with symptoms including abdominal pain, fever, malaise, cramping and severe and/or bloody diarrhea that generally last anywhere from 7 to 14 days [6]; in rare cases infection with enteric pathogens can result in more severe illness such as Guillan-Barré syndrome, reactive arthritis or haemolytic uremic syndrome (HUS) [7]. Incubation times vary between viral, bacterial and parasitic pathogens being as short as 24 h for some virus (Norwalk-like virus) to several weeks with some parasitic pathogens [6].

Understanding spatial and temporal patterns in disease incidence can give an indication to the etiology of the disease. Seasonal patterns in enteric disease incidence have been seen with infection with *E. coli* O157, campylobacteriosis, giardiasis, salmonellosis and shigellosis [8–15]. Seasonal patterns have been thought to occur, in part, due to social and behavioural factors such as barbequing during the summer season [16] or outdoor recreational activities such as swimming or camping [13, 14]. Other hypotheses relate disease incidence with contamination of local water supplies from agricultural or environmental sources [17, 18]. Several studies have demonstrated an association between weather events and disease incidence. Variations in the spatio-temporal distribution of disease incidence could be related to weather such as increases in temperature [10, 19, 20] or increased precipitation [21]. Lastly, seasonality may be linked to infections acquired while travelling [16, 22]. Several studies have found links between the spatial distribution of enteric disease incidence and agricultural activities [15, 23–25].

Although several studies have examined the spatial and temporal distribution of *E. coli* O157 and *Giardia* in Ontario, Manitoba and Alberta [11, 14, 15, 23, 25], the spatial distribution of enteric diseases has not been well characterized for New Brunswick. The objectives of this study were to describe the spatial and temporal distribution of the incidence of reportable *Campylobacter*, *E. coli*, *Giardia*, *Salmonella* and *Shigella* in New Brunswick, Canada.

## Methods

### Case data

Laboratory confirmed case counts, as defined by New Brunswick provincial reportable disease system, for *Campylobacter* spp., *Escherichia coli*, *Giardia* spp., *Salmonella* spp. and *Shigella* spp. were obtained from the New Brunswick Department of Health and Wellness for the time period January 1, 1994 to December 31, 2002. Patient identification numbers had been removed from the data and a sequential identification number added to preserve patient confidentiality and to meet the University of Guelph’s Research Ethics Board Guidelines for the use of secondary data sources. The data provided contained information on the age, sex, reporting date, diagnosis date and postal code of the case. Due to privacy issues, full postal code data was unavailable after 2002, as such, data after this year was not included in the study.

A large number of cases were removed due to incomplete or missing postal codes which were needed to link case information to census subdivisions (CSD) or watershed (WS) identifiers for purposes of aggregation (Table 1). Other cases were removed because the provided postal code could not be mapped to known New Brunswick watershed or CSD areas (i.e. postal code information was complete but was geographically mapped outside of New Brunswick provincial boundaries). Other reasons why cases were removed included incomplete date information, invalid postal code and large discrepancies between admission date and reporting date. A summary of the number of cases used in the study can be seen in Table 1. The majority of the *Escherichia coli* cases were of the O157 serotype, with only 2.8 % (6/215) being non-O157. For the purposes of this study, where *E. coli* cases are referred to, they will be referred as *E. coli* thus encompassing both O157 and non-O157 strains.

**Table 1:** Case counts of reportable enteric pathogens used based on reclassification of reportable pathogenic organisms, based on species, and valid postal code information for New Brunswick, Canada from 1994-2002

Classification	Original classification	Number of cases	Total number of cases	Number used in study
*Campylobacter* spp.	Campylobacteriosis	2319	2319	1460
*Escherichia coli*	*Escherichia coli* enteritis: other	6	215	99
	*Escherichia coli*	209		
*Giardia* spp.	*Giardia*sis	903	903	528
*Salmonella*	*Salmonella* enteritis	1245	1262	801
	*Salmonella paratyphi* enteritis	3		
	*Salmonella typhi* enteritis	14		

To assess the temporal distribution of the 5 different pathogens, case counts, for each pathogen, were summed to create weekly and monthly case counts for the entire province. Week was defined as the week of the year (1–52) in which reporting occurred, or the month of the year (1–12) in which reporting occurred. These were used to estimate the incidence rate for each pathogen respectively using population estimates from the 1996 population census [26] in the denominator.

For spatial analysis, case counts were calculated for the two spatial scales (CSD and WS) that would be used in the spatial analysis. The postal code field contained in the reportable disease dataset obtained from New Brunswick Health and Wellness was used to link data from that dataset to CSD and WS identifiers (see "Population Data for Spatial Analysis" below) and population estimates. All cases in a given geographical area were summed over the duration of the study period. The case counts were then used to estimate incidence rates for each area using the population estimate for that area (see below) and expressed on a yearly basis (cases per person per year).

### Time series

Monthly incidence rate time series were plotted for each reportable disease. A moving average for 3 month (seasonally) and 13 month (yearly) smoothed incidence rates were estimated. These estimates were plotted in conjunction with the raw monthly incidence rates.

### Long term trends

Negative binomial regression was used to test the significance of long term trends by examining monthly incidence rates over the length of the study period [27]. A negative binomial distribution was used because in several cases disease incidence exhibited significant overdispersion, thus a Poisson distribution would not be appropriate. Month of study was used as an explanatory variable of monthly cases of disease to control for seasonal and holiday effects related to month. Population estimates of New Brunswick were obtained from the 1996 Canadian Census and were used as the exposure offset for modelling purposes.

### Seasonal decomposition

Seasonal decomposition of monthly incidence rates was performed using the data driven LOESS smoothing method outlined in [28]. Monthly incidence was used for the seasonal decomposition to minimize noise present in the time series plots. The temporal scan statistic developed by [29] was used to examine the data for temporal clusters of disease. A purely temporal scan statistic gives an indication into the most likely temporal clusters of disease. Each year of the data series was examined for the occurrence of peak disease incidence. A Poisson distribution was used with a maximum temporal cluster size of 60 days. Kulldorff [30] recommends a maximum temporal cluster size that is 50 % of the length of the study period. That would represent a maximum temporal cluster size of 183 days as the test was conducted on each year of the data. A maximum temporal cluster size that was smaller than recommended value was used because the objective was to identify relatively short periods of time that may give an indication of seasonality of disease incidence. Data manipulation was carried out using Stata version 9.1 [31]. Graphing, moving average calculations and seasonal decomposition were performed in R [32]. Temporal clusters of disease were determined using the cluster detection software package SaTScan [30].

### Population data for spatial analysis

An enhanced postal code file was used to determine estimates of the population for each geographical area using population values for each postal code based on the 1996 census of population [33]. Postal codes were linked to CSD and WS information using a spatial join in ArcGIS [34]. A postal code was considered to associated with a CSD or WS if the center of the postal code inside the boundary of the CSD or WS. The population for each geographical region was estimated by summing the population estimates of all postal codes in the geographical area. Age and sex distribution was not available for each postal code, therefore population estimates for age and sex strata could not be determined. This prevented the estimation of age and sex adjusted rates for each CSD/WS.

### Empirical bayes smoothing

Empirical Bayes (EB) estimates were calculated for incidence rates at both the CSD and WS geographical scales [35]. Mapping and estimation of EB smoothed incidence rates was performed using the spatial dependence (spedep) library [36] of the R software package [32].

### Spatial clustering and cluster detection

Moran’s I was used to examine spatial clustering of the incidence rates. Specifically Moran’s I was applied to the EB smoothed incidences, because EB smoothing is a kind of spatial standardization with respect to varying sample sizes. Furthermore, the Queen contiguity structure was specified, which connects each region with all its direct neighbours [37].

The spatial scan statistic [29] was used to examine raw case counts for clusters of disease. Raw, rather than smoothed rates, were also used with the spatial scan statistic. Data were examined for high disease rate clusters using pure spatial clusters based on a Poisson model with a maximum geographical cluster size of 20 % of the population at risk. A maximal geographical cluster size of 20 % of the population was chosen as enteric diseases are rare in the population and it is unlikely that more than 20 % of the population would be at risk in a single outbreak. Since enteric disease outbreaks tend to be governed by localized risk factors and disease processes, only a fraction of the population should be affected. The optimal parameter setting for the maximum cluster size is often not clear [38]. Adjustments were made to allow for testing of significance of secondary clusters [39]. SaTScan software [30] was used to identify and test significance of spatial clusters. Statistical methodology for the spatial scan statistic has been described elsewhere [40]. Significant spatial clusters identified by the spatial scan statistic were mapped using ArcGIS [34].

### Disease mapping

Census subdivision shapefiles were obtained from Statistics Canada and watershed boundary shapefiles were obtained from Environment Canada. Choropleth maps of EB smoothed incidence rates were mapped using ArcGIS [34].

## Results

### Summary statistics

Summary statistics for weekly incidence rates are presented in Table 2 and indicate that *Campylobacter* was the most commonly reported enteric pathogen infection in New Brunswick based on the median and mean incidence. *Salmonella* and *Giardia* infections were second and third most commonly reported enteric pathogens, respectively. *E. coli* and *Shigella* infections had the lowest median incidence of reported cases. Reportable *E. coli* infections had a higher mean incidence than reported *Shigella* infections.

**Table 2:** Summary statistics and trend results from negative binomial regression examining reportable enteric disease incidence (1994 to 2002) in New Brunswick, Canada

	Mean^1^	Median^1^	Range^1^	Beta coefficient for trend
*Campylobacter*	1.831	1.761	0.271-5.419	0.005 (*p* > 0.001)
*Escherichia coli*	0.124	0	0-1.897	0.026 (*p* = 0.001)
*Giardia*	0.662	0.677	0-1.897	0.002 (*p* = 0.220)
*Salmonella*	1.005	0.948	0-2.980	0.003 (*p* = 0.039)
*Shigella*	0.039	0	0-0.270	0.008 (*p* = 0.001)

^1^cases per 100,000 person-week

### Time series

**Fig. 1:**
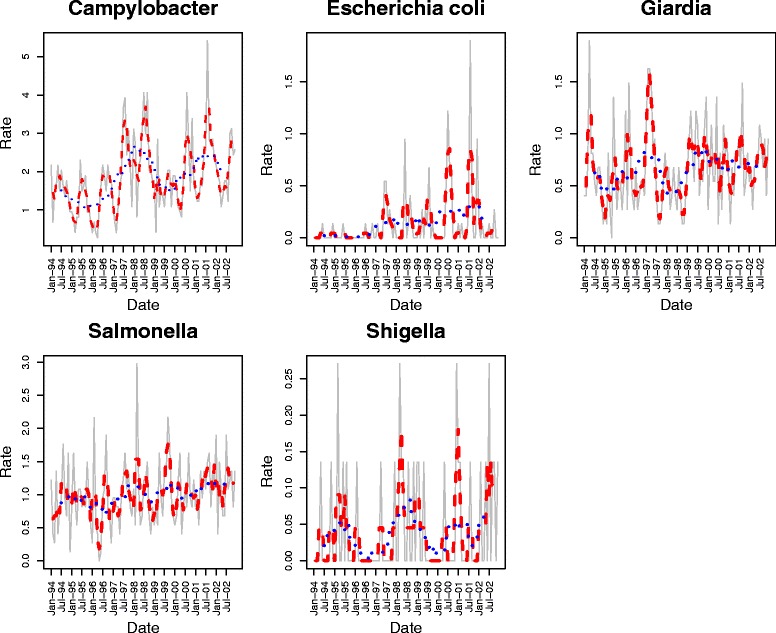
Monthly incidence of reportable enteric illness (1994–2002) in New Brunswick, Canada. Rates are expressed in cases per 100,000 person-month. Data are presented as 2 month moving averages (red-dashed) and 13 month (blue-dashed) moving averages

Time series plots of reportable *Campylobacter*, *E. coli*, *Giardia*, *Salmonella* and *Shigella* can be seen in Figure 1. Except for reportable *Shigella* infections, all time-series plots exhibited a marked seasonal component based on 3 month moving average values. Several peaks were seen throughout the year with the majority of the peaks occurring in winter and spring.

### Long term trends

Negative binomial regression was performed for each of the disease outcomes to determine the significance of any secular trend. Table 2 reports the estimate of the slope parameter that represents the trend in the time series of reportable enteric pathogens. The incidence rate of *Campylobacter*, *E. coli*, *Salmonella* and *Shigella* infections reported in New Brunswick all exhibited a significant increasing secular trend (Table 2). The incidence of reportable *Giardia* infections did not exhibit a trend in time (Table 2).

### Seasonal decomposition

**Fig. 2:**
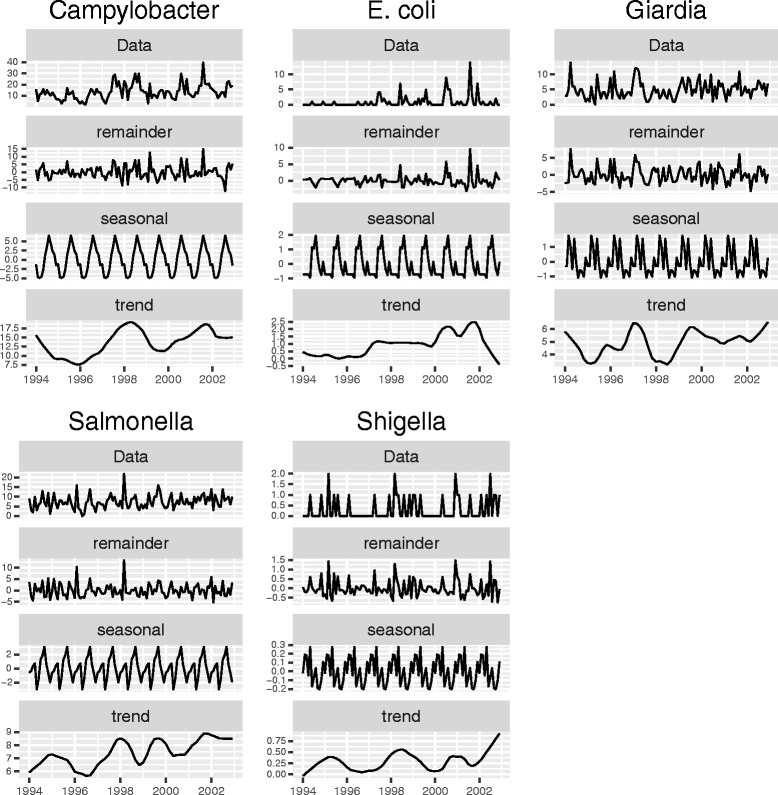
Seasonal decomposition of the monthly incidence reportable enteric illness (1994–2002) in New Brunswick, Canada

Seasonal decomposition of reportable enteric disease rates are shown in Fig. 2. The incidence of *Campylobacter* infections peaked in early summer. The incidence of human *E. coli* infections showed a bi-modal seasonal pattern with the largest peaks occurring in the summer and a smaller peak occurring January. Several peaks were observed in the seasonal decomposition of *Giardia* disease rates. Two large peaks were observed in winter and spring. A smaller peak can be seen in late autumn. Seasonal decomposition of the incidence of *Salmonella* infections indicated a bi-modal seasonal pattern with the peak incidence occurring during the summer months with a peak about half the size occurring in spring. No discernable seasonal pattern could be extracted from the *Shigella* incidence.

### Temporal scan statistic

Primary temporal clusters identified by the temporal scan statistic are presented in Table 3 for each year of the study period for each disease outcome. Six disease clusters were identified for the incidence of *Campylobacter* infections. All clusters occurred in the summer months (July, August, and September). There were five temporal clusters for the incidence of *E. coli* infections. Clusters occurred in late spring extending into the summer months. Three temporal clusters of the incidence for *Giardia* infections were identified and all occurred in spring. Five temporal clusters identified for the incidence of reportable *Salmonella* infections. The temporal distribution of these disease clusters was more varied than with the other pathogens. Two temporal clusters were identified in the fall (October, November, and December) and one in each of the winter, spring and summer. No temporal clusters were identified for the incidence of *Shigella* infections.

**Table 3:** SaTScan temporal clusters reportable enteric disease (1994 to 2002) in New Brunswick, Canada

Year of study	*Campylobacter*	*Escherichia coli*	*Giarida*	*Salmonella*	*Shigella*
1993	--	--	--	--	--
1994	May 5 – May 21	None found	Apr 3 – May 21^a^	Nov 13 – Nov 26^a^	None found
1995	Jul 24 – Sep 17^a^	None found	May 29 – Jun 11^a^	Feb 6 – Mar 5	Mar 20 – Mar 26
1996	Jun 19 – Jul 9^a^	None found	Mar 6 – Apr 2^a^	Jul 28 – Sep 17^a^	None found
1997	Jul 24 – Aug 27^a^	Jun 12 – Aug 6^a^	Mar 13 – Apr 23	Aug 14 – Aug 20	None found
1998	Jul 7 – Aug 13^a^	May 29 – Jun 18^a^	Apr 24 – May 21	Mar 13 – Apr 9^a^	Mar 27 – Apr 9
1999	Mar 6 – Mar 26	Aug 7 – Aug 20^a^	Mar 27 – Apr 2	Sep 25 – Oct 15^a^	Jan 16 – Feb 26
2000	Jul 31 – Aug 20^a^	Jun 12 – Aug 6^a^	Feb 28 – Mar 5	Mar 27 – Apr 4	Dec 4 – Dec 24
2001	Jul 31 – Sep 24^a^	Aug 7 – Aug 27^a^	Aug 28 – Oct 1	Nov 27 – Dec 10	Jan 2 – Feb 5
2002	Nov 27 – Dec 3	Oct 9 – Oct 22	Jul 17 – Jul 30	Jan 23 – Jan 29^a^	Jul 10 – Jul 30

-- Data not available

^a^Indicates a significant cluster at the 95 % confidence level

### Spatial distribution of empirical Bayes estimates

**Fig. 3:**
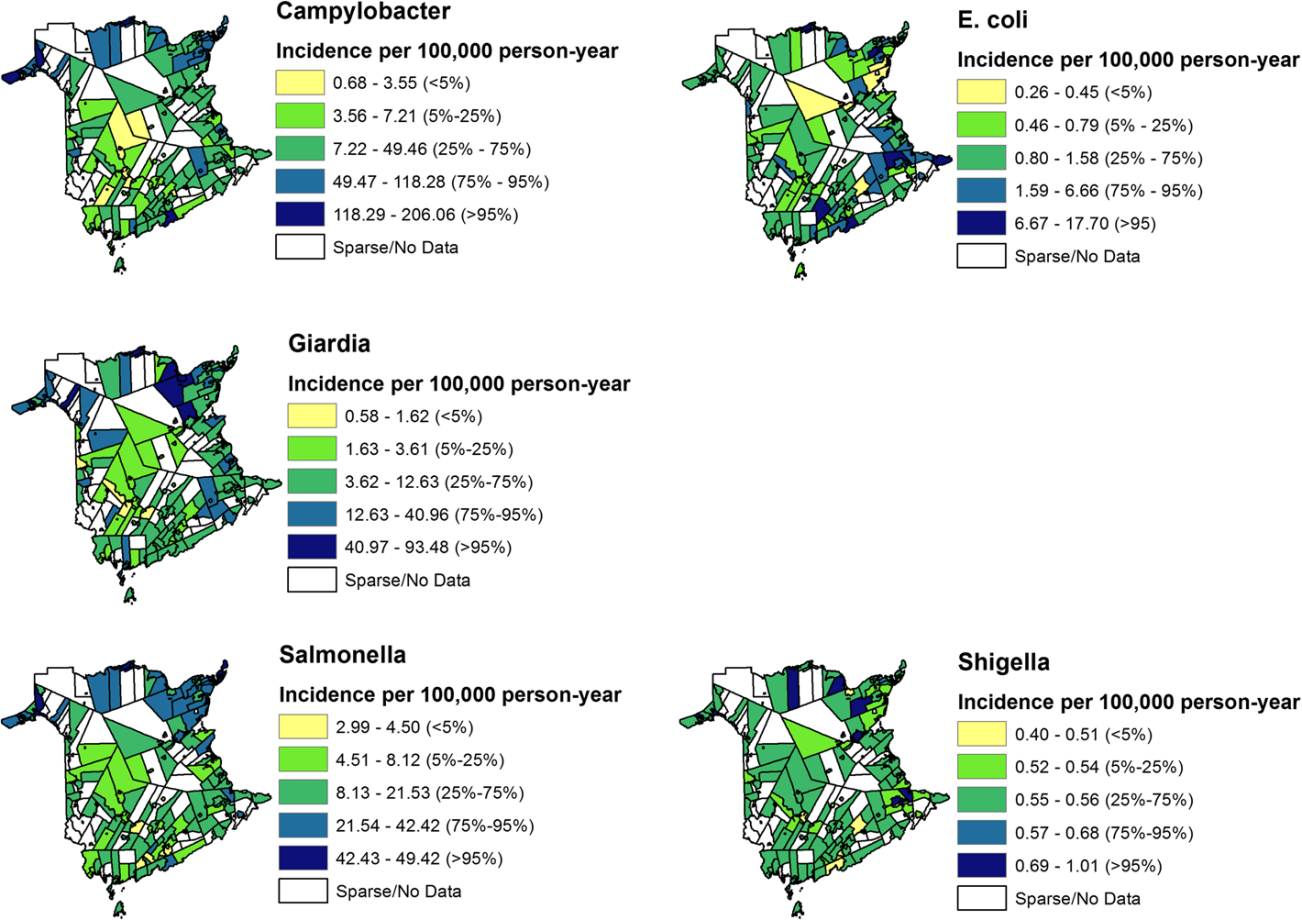
Empirical Bayes smoothed incidence rates for reportable enteric illness (1994–2002) in New Brunswick, Canada at the Census Subdivision level (CSD). Darker areas indicate areas of higher disease incidence

Choropleth maps of the EB smoothed yearly incidence of reportable *Campylobacter*, *E. coli*, *Giardia*, *Salmonella* and *Shigella* for CSD areas of New Brunswick can be seen in Fig. 3. Upon visual inspection, there appears to be some grouping of areas with high incidence, with the exception of *Shigella* infection rates. The spatial distribution of EB smoothed incidence rates of reported *Campylobacter* infections in CSD areas was highest in the middle of the province, with other potential areas of high incidence in the west and east of the province. The incidence of EB smoothed *E. coli* infections in New Brunswick CSD areas were evenly distributed throughout the province with a possible grouping of high incidence areas in the north-east corner of the province. Spatial distribution of the EB smoothed incidence rates of reported *Giardia*, *Salmonella* and *Shigella* infections at the CSD level were evenly distributed throughout the province.

**Fig. 4:**
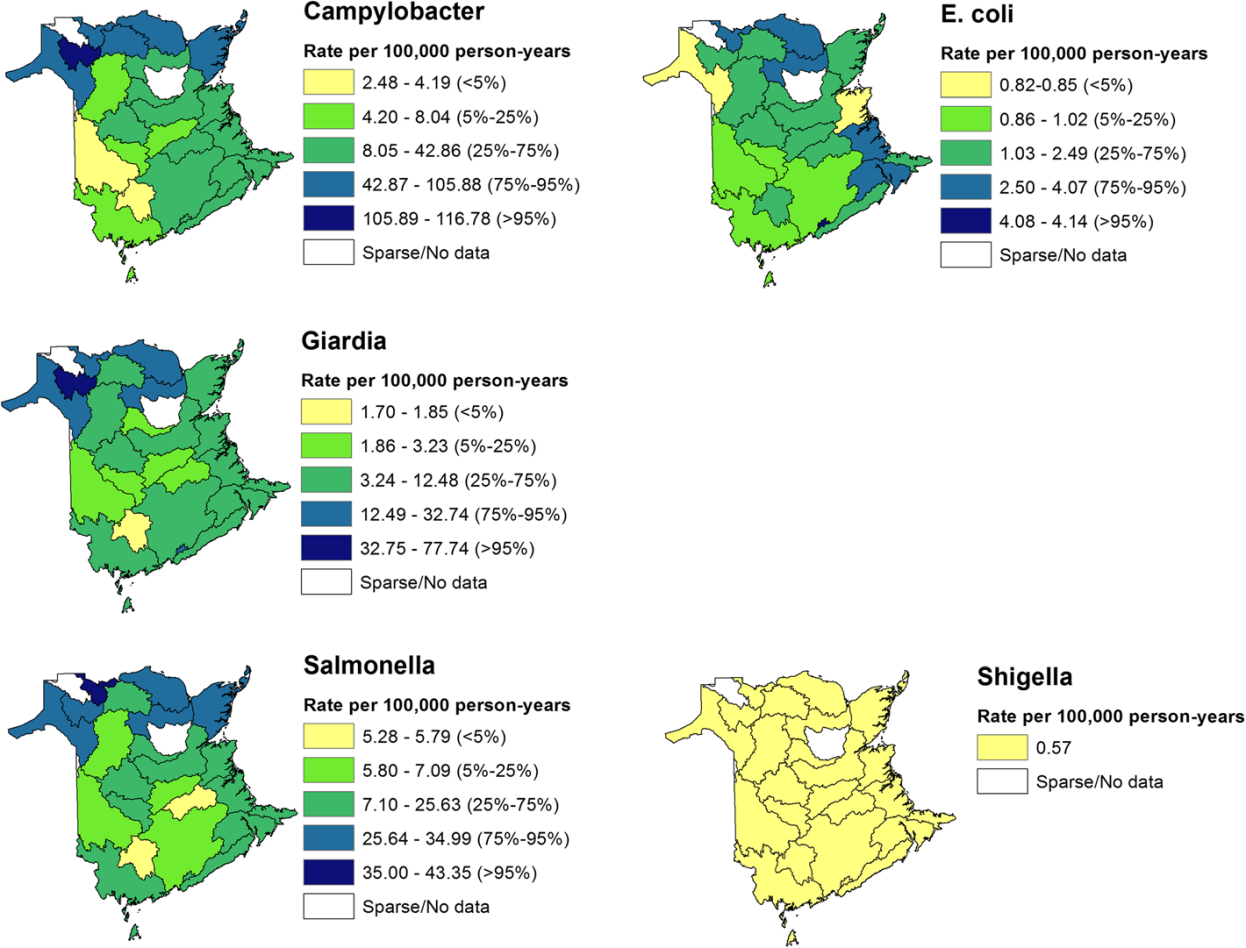
Empirical Bayes smoothed incidence rates for reportable enteric illness (1994–2002) in New Brunswick, Canada at the watershed level (WS). Darker areas indicate areas of higher disease incidence

Watershed based maps of the yearly incidence rates of reportable *Campylobacter*, *E. coli*, *Giardia*, *Salmonella* and *Shigella* are shown in Fig. 4. Higher incidences rates of reported *Campylobacter* infections can be seen in the north area of the province with a possible grouping of high incidence areas in the north-west. *E. coli* incidence rates were highest in the north, central and south-east areas of the province. EB smoothed *Giardia* rates were evenly distributed throughout the province with areas of higher incidence located in the north-west corner of the province. The spatial distribution of EB smoothed reported *Salmonella* incidence rates was evenly distributed through the province with the possible exception in the north of the province where areas of higher incidence were grouped. Visual inspection of EB smoothed *Shigella* incidence rates revealed no discernable spatial pattern.

### Moran’s I

Moran’s I and associated *p*-values for the incidence of enteric illness in New Brunswick are summarized in Table 4. Positive spatial autocorrelation was observed at both the CSD and watershed levels for the incidence of *Campylobacter*, *Giardia* and *Salmonella* infections. No evidence was found for autocorrelation within the incidence of *Shigella* and *Escherichia coli* infections at the CSD level. Moran’s I for *E. coli* at the watershed level was not significant indicating that there was no spatial autocorrelation present.

**Table 4:** Moran’s I as calculated by ArcGIS (version 9.1) reportable enteric illness (1994–2002) in New Brunswick, Canada

Disease	Geographical scale	Moran’s *I*	*p*-value
*Campylobacter*	CSD	0.2031	0.004^*^
	Watershed	0.4693	0.001^*^
*Escherichia coli*	CSD	0.1897	0.010^*^
	Watershed	0.1419	0.056
*Giardia*	CSD	0.3467	0.002^*^
	Watershed	0.1635	0.019^*^
*Salmonella*	CSD	0.3946	0.001^*^
	Watershed	0.3696	0.002^*^
*Shigella*	CSD	0.0280^*^	0.240
	Watershed	0.2913	0.012^*^

*Significant at the 95 % confidence level

### Spatial scan statistic

**Fig. 5:**
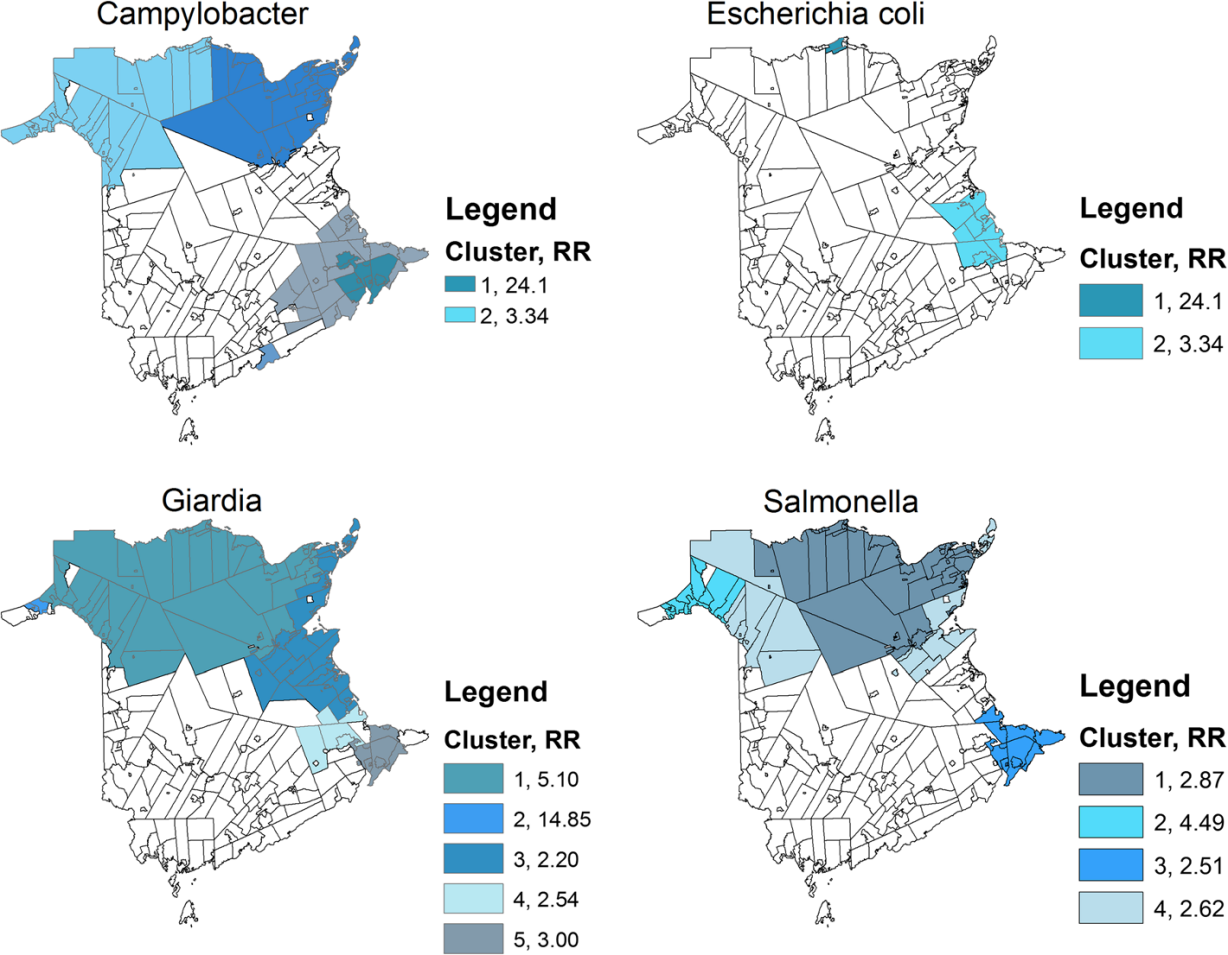
Significant spatial clusters (*p*-value < 0.05) for reportable enteric illness (1994–2002) in New Brunswick, Canada at the Census Subdivision level (CSD) as identified by the spatial scan statistic

**Fig. 6:**
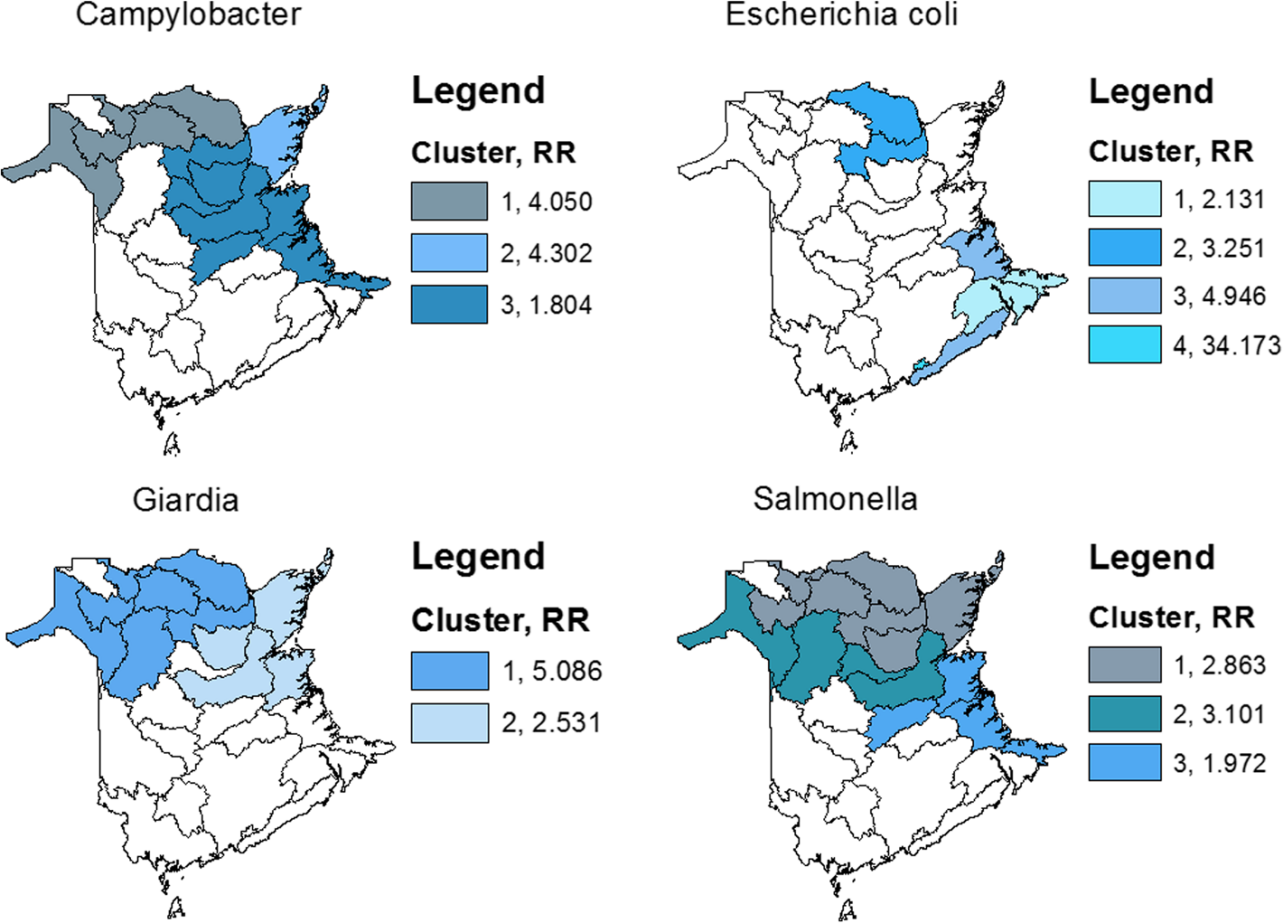
Significant spatial clusters (*p*-value < 0.05) for reportable enteric illness (1994–2002) in New Brunswick, Canada at the watershed level (WS) as identified by the spatial scan statistic

Maps showing the results of the spatial scan statistic can be seen in Fig. 5 for CSD level data and Fig. 6 for WS level data and Tables 5 and 6 summarize the findings for the CSD and WS levels, respectively. The relative risks represent the risk of disease inside the cluster (i.e. the exposed population) over the risk of disease outside the cluster (the unexposed population). Spatial clusters in the incidence of enteric diseases were observed for all pathogens being studied at both the CSD and WS geographical scales, with the exception of the incidence of *Shigella* infections where no significant spatial clusters were detected at either the CSD or WS level. Exact locations of spatial clusters of high incidence are shown in Figs. 5 and 6. Relative risk for spatial clusters of reported enteric pathogen infections at the CSD level ranged from 2.2 to 54.8, depending on the pathogen (Table 5); smaller relative risks were observed at the WS level with relative risks ranging from 1.8 to 34.2 (Table 6).

**Table 5:** Significant clusters of reportable enteric disease incidence in New Brunswick, Canada at the Census Subdivision (CSD) level as identified by the spatial scan statistic

Pathogen	SaTScan cluster number	*p*-value	Relative risk
*Campylobacter*	1	0.001	4.322
	2	0.001	3.834
	3	0.001	2.726
	4	0.001	2.890
	5	0.001	54.792
*E. coli*	1	0.001	24.102
	2	0.001	3.337
*Giardia*	1	0.001	5.100
	2	0.001	14.845
	3	0.001	2.202
	4	0.001	2.544
	5	0.004	3.002
*Salmonella*	1	0.001	2.865
	2	0.001	4.490
	3	0.001	2.512
	4	0.001	3.052
*Shigella*	None Found

**Table 6:** Significant clusters or reportable enteric disease incidence in New Brunswick, Canada at the watershed level (WS) as identified by the spatial scan statistic

Pathogen	SaTScan cluster number	*p*-value	Relative risk
*Campylobacter*	1	0.001	4.050
	2	0.001	4.302
	3	0.001	1.804
	4	0.047	3.027
*E. coli*	1	0.027	2.131
	2	0.004	3.251
	3	0.002	4.946
	4	0.013	34.173
*Giardia*	1	0.001	5.086
	2	0.001	2.531
*Salmonella*	1	0.001	2.863
	2	0.001	3.101
	3	0.001	1.972
*Shigella*	None Found		

## Discussion

The etiology of enteric disease is not well understood with a number of factors influencing disease incidence, including factors that vary in time and space. Risk factors for enteric disease have been examined primarily in central and western Canada, with little research having been conducted in the Atlantic region of the country. The objective of this study was to examine the spatio-temporal distribution of reportable enteric diseases in New Brunswick to gain further insights into the factors that influence enteric disease incidence.

### Geographical impacts

The spatial scan statistic identified that cases of enteric disease cluster. The general location and size of the reportable disease clusters detected by the spatial scan statistic did not differ greatly when comparing analyses from the CSD level (Fig. 5) to that of watershed level (Fig. 6). Previous studies have observed that the specification of a finer geographic resolution did not improve sensitivity of the scan test to detect variations in risk of disease (i.e. to detect disease clusters) [41]. The agreement between the results from both geographic scales strengthens the hypothesis that a possible common exposure contributes to the incidence of reportable enteric disease in New Brunswick; disagreement between the two geographical scales may be a result of the modifiable areal unit problem (MAUP), which is a consequence of aggregating data across different geographical scales [42, 43].

Aggregation of data to a higher geographical level provided a more stable estimate of disease rates as denominators were larger and the addition of a small number of cases (1–2 cases) did not greatly affect the estimated rate for that area. In several cases, the denominators for the CSD level data were very small or zero. This was less of a problem when data were expressed on the watershed level. Differences in the spatial pattern of disease incidence between CSD and WS levels were expected, due to the differences in population size between CSD and WS areas. The two levels of aggregation are also distinct in that CSD areas are political boundaries, whereas watershed boundaries follow natural geographical features. CSD areas are convenient from a resource allocation perspective, but underlying environmental exposure patterns typically do not follow such political boundaries. By examining disease incidence according to more naturally occurring boundaries, potential environmental exposures may be explored more appropriately.

Interpretation of EB smoothed maps was complicated by the nature of the CSD areas where rural areas tend to be represented by large geographical areas, which visually overwhelm urban areas that are represented by smaller geographical areas. In these cases, the larger areas provide a greater visual stimulus, more so than the smaller areas, thus attracting the eye. The use of choropleth maps in mapping health data provided a minimal level of information about the spatial distribution of disease incidence; their use is limited due to the problems outlined above.

There are a number of potential disease pathways that may contribute to the clustering of enteric illness incidence. Large municipalities in New Brunswick, as well as some incorporated areas, have municipal water supplies that draw water from surface and ground sources. These regions employ water treatment and distribution systems [44]. Rural areas, on the other hand, which account for about 40 % of the population (approximately 300,000 persons), primarily source their water from private wells [44]. These wells are prone to contamination and are not regularly tested [44]. Studies have identified living in rural areas as a risk factor for enteric illness [13, 15]. Small towns and rural areas may have lacked the necessary water treatment facilities to effectively treat water against contamination with enteric pathogens.

Livestock rearing and manure management practices may be contributing to the spatial distribution of enteric disease incidence. Studies in Ontario and Alberta have identified living in proximity to livestock and agricultural land treated with manure [15, 45–47] as risk factors for infection. Not all pathogens examined in this study have previously demonstrated associations with agriculture. Odoi et al. [25] did not find an association between the incidence of reported *Giardia* infections and livestock density in Ontario. Only about 5 % of New Brunswick’s area is dedicated to agricultural land [48]. The effect of agriculture on enteric disease incidence in New Brunswick is not well understood. Further investigation is required, particularly in light of the fact that agricultural land use is limited and that agriculture alone does not explain the variation in enteric disease incidence [49].

Forestry is the primary industry of New Brunswick and approximately 80 % of New Brunswick’s land mass is productive forest [50]. Little, if anything, is known about the impact of forestry practices on the incidence of enteric illness. The harvesting of lumber has been shown to increase the rate of run-off from the land, increase stream flow, and increase the rate and timing of snow melt [51]. Increased stream flows have been associated with increased risk of flooding [51]. This has the potential to contaminate local surface water supplies, or contaminate improperly maintained or damaged wells.

### Seasonal peaks in disease incidence

Seasonal time series decomposition of *Giardia* incidence identified peaks that have previously been reported [13, 14], but also identified seasonal fluctuations in disease incidence that have not been reported before. In particular, the spring peak for *Giardia* did not follow the pattern observed in other Canadian studies of *Giardia* infections. Studies conducted in two Canadian provinces [13, 14, 52], Ontario and Alberta, and the United States [8] have reported peaks in *Giardia* incidence during the late summer to early fall. Seasonal Decomposition of *Salmonella* incidence and the temporal clusters of disease identified seasonal peaks that agree with the seasonal patterns reported by Guerin et al. [17], who reported peak disease incidence as occurring in July to September; although a report from Ontario indicated peak incidence in the spring [9] which was also observed in this study. The seasonality of other bacterial pathogens in the study is consistent with previously reported findings. Both the seasonal decomposition and the temporal scan statistic indicate that peak incidence occurred during the summer months. Previous studies have demonstrated peak incidence for *E. coli* and *Campylobacter* infections during the spring and summer months [15, 16, 19, 53–55].

There are a number of potential risk factors that may play a role in disease incidence and explain the observed peaks in enteric disease incidence. Among these are natural weather patterns and agricultural activities. Weather has been identified as a factor in triggering waterborne disease outbreaks, primarily related to excess rainfall [3, 56–58]. A study of waterborne outbreaks in Canada revealed that outbreaks peaked during the spring/summer and that these were primarily related to meteorological conditions or extreme weather events [59]. The peak incidence of *Giardia* and the smaller secondary peak incidence seen for *Salmonella* infections possibly correspond with peak run-off from spring melt. Atherholt et al. [60] observed increased level of *Giardia* cysts in river water following snow melt events. Spring snowmelt in New Brunswick peaks in mid-April to May and is responsible for the largest stream flow volumes [61]. This is in contrast to Ontario and Alberta, which get approximately half the amount of snow annually [62]. The timing of spring thaws for Ontario and Alberta also differs from New Brunswick with Ontario’s thaw occurring in March and Alberta having a much more variable winter with thaws throughout the season [62].

A number of studies have demonstrated the role of agriculture in enteric illness [15, 21, 45, 54]. Agriculture may impact disease rates through a variety of different routes. Direct contact with livestock or run-off from agricultural land has been identified as a risk factor for infections with *Campylobacter*, *E. coli* O157 and *Salmonella* [2, 54, 57, 63, 64].

Peaks in disease incidence could be related to the occurrence of outbreaks, as these were not adjusted for in the analysis. An examination of outbreaks during the study period (data not shown) identified several outbreaks that coincided with identified temporal clusters of disease. While this explains some of the temporal clustering of *Salmonella* cases, there did not appear to be any reported outbreaks in the literature that could explain the observed temporal clustering of *Giardia* cases.

The spring peaks observed in the incidence of *Giardia* and *Salmonella* infections in New Brunswick could be due to unmeasured factors. However, they may also be due to a different etiological pathway in New Brunswick compared to Ontario and Alberta. This study was unable to assess the effects of differences in population dynamics (e.g. age, sex, socio-economic factors), but the age and sex population distributions in New Brunswick are reflective of the national distribution [65]. This gives an indication that these unmeasured factors may be environmental in nature (e.g. geography or climate) as opposed to demographic factors [60].

### Long term trends

Reportable enteric disease incidence rates appeared to be increasing in New Brunswick over the time period studied. Four of the five pathogens exhibited a linear increase in disease incidence over the study period (Table 1). The incidence rate of *Giardia* infections was the only disease that remained stable over the study period. This differs from trends seen at the National level [1]. Possible explanations for the increase in New Brunswick of incidence rates of *Campylobacter*, *E. coli*, *Shigella* and *Salmonella* include a true increase in the incidence of these infections over time or that there may also be an increase in the reporting of these diseases over the study period causing an apparent increase in disease incidence. The study period was also relatively short and this could have an impact on the temporal trends observed. An annual report by the New Brunswick Department of Health and Wellness, published in 2013 [66], indicated that *Campylobacter* and *E. coli* incidence rates have been stable since 2003; whereas reported *Giardia, Salmonella* and *Shigella* infections have increased slight in incidence. The differences observed between this study and current rates of disease may reflect difference in analytical techniques and temporal scales used to examine long term trends in the data.

### Limitations

Reportable disease data under represent the true number of cases in the population. It has been estimated for Ontario, Canada that for every reported case of enteric illness, 313 cases are not captured by the system [5]. This has the potential to introduce bias into the study if the population captured by the reporting system differs significantly from the New Brunswick population. It was not possible to remove outbreak-associated cases of enteric illness from the database, as information pertaining to whether a case arose due to an outbreak was not recorded in the database used. This could potentially impact the results of the spatial scan statistic since cases arising from an outbreak tend to cluster spatially. A large number of cases were removed from the data analyzed due to missing data or our inability to link cases to a spatial region, which may introduce bias if those cases are related to some spatial or temporal factor. Bias could be a problem if these cases differ significantly from the New Brunswick population that they represent. Lastly, it was not possible to distinguish between cases arising from foodborne, waterborne or person-to-person transmission. The etiology of disease incidence in the cases may be different and exhibit different spatial and temporal patterns.

## Conclusions

This is the first time that the spatial pattern of enteric illness in New Brunswick has been investigated. This study identified several clusters of enteric disease in the province. The spatial distribution of enteric illness in New Brunswick was examined at two different geographical scales. While the results from each analysis generally agreed, it was not clear which choice of geographical scale was best for analyzing public health data. More research is needed to explore the impact of scale choice in the analysis of spatial and temporal data; particularly for geographical areas that are predominately rural and where population density is sparse.

This study identified spring peaks in disease incidence for several enteric pathogens. Outbreaks may explain some of the peaks in observed disease incidence, but they do not account for all of the variation seen. We also observed that the incidence of *Campylobacter*, *E. coli*, *Salmonella* and *Shigella* infections exhibited a significant increasing trend over the time period under study. This differed from disease incidence at the national level and from what was observed in subsequent years in New Brunswick. The use of a finer temporal scale may help identify trends in disease incidence that are masked by coarser measures of disease incidence otherwise. Additionally, the use of seasonal decomposition techniques and the temporal scan statistic allowed for the identification of seasonal patterns of disease incidence not previously reported. The combination of these statistical procedures in conjunction with traditionally used methods might help identify temporal trends in enteric disease incidence that are of public health importance.

The findings from this study indicate a need for high quality data that provides sufficient temporal and spatial resolution to allow for a high level of analysis. In this study, we identified novel spatial and temporal trends in enteric disease incidence and postulated potential disease etiologies. Further research is needed to determine if forestry, wildlife, agriculture, urbanization, and socio-economic factors are associated with the spatial and temporal variation in disease rates.

## Abbreviations

CSD: Census sub-division
EB: Empirical Bayes
WS: Watershed

## References

[1] Public Health Agency of Canada (2014). National enteric surveillance program (NESP) annual summary 2012.

[2] Stirling R, Aramini J, Ellis A, Lim G, Meyers R, Fleury M (2001). Waterborne cryptosporidiosis outbreak, North Battleford, Saskatchewan, spring 2001. Can Commun Dis Rep.

[3] Auld H, MacIver D, Klaassen J (2004). Heavy rainfall and waterborne disease outbreaks: the Walkerton example. J Toxicol Environ Health A.

[4] Aramini J, Willson J, Allen B, Sears W, Holt J, McLean M (2000). Water quality and health care utilization for gastrointestinal illness in greater Vancouver.

[5] Majowicz SE, Doré K, Flint JA, Edge VL, Read S, Buffett MC (2004). Magnitude and distribution of acute, self-reported gastrointestinal illness in a Canadian community. Epidemiol Infect.

[6] Hunter PR (1997). Chapter 3: drinking water and waterborne disease. Waterborne disease: epidemiology and ecology.

[7] Hunter PR (1997). Chapter 15: camplyobacteriosis. Waterborne disease: epidemiology and ecology.

[8] Mohamed AS, Levine M, Camp Jr JW, Lund E, Yoder JS, Glickman LT, et al. Temporal patterns of human and canine *Giardia* infection in the United States: 2003–2009. Prev Vet Med. 2014;113:249–56. doi:10.1016/j.prevetmed.2013.11.006. 10.1016/j.prevetmed.2013.11.006 PMC11307260 24309130

[9] Varga C, Pearl DL, McEwen S, Sargeant JM, Pollari F, Guerin MT. Incidence, distribution, seasonality, and demographic risk factors of *Salmonella enteritidis* human infections in Ontario, Canada, 2007–2009. BMC Infect Dis. 2013;13:212. 10.1186/1471-2334-13-212 PMC3655886 23663256

[10] Fleury M, Charron DF, Holt JD, Allen OB, Maarouf AR (2006). A time series analysis of the relationship of ambient temperature and common bacterial enteric infections in two Canadian provinces. Int J Biometeorol.

[11] Pearl DL, Louie M, Chui L, Doré K, Grimsrud KM, Leedell D (2006). The use of outbreak information in the interpretation of clustering of reported cases of *Escherichia coli* O157 in space and time in Alberta, Canada, 2000–2002. Epidemiol Infect.

[12] Gupta A, Polyak CS, Bishop RD, Sobel J, Mintz ED (2004). Laboratory-confirmed shigellosis in the United States, 1989–2002: epidemiologic trends and patterns. Clin Infect Dis.

[13] Odoi A, Martin SW, Michel P, Holt J, Middleton D, Wilson J (2003). Geographical and temporal distribution of human giardiasis in Ontario Canada. Int J Health Geogr.

[14] Greig JD, Michel P, Wilson JB, Lammerding AM, Majowicz SE, Stratton J (2001). A descriptive analysis of giardiasis cases reported in Ontario, 1990–1998. Can J Public Health.

[15] Michel P, Wilson JB, Martin SW, Clarke RC, McEwen SA, Gyles CL (1999). Temporal and geographical distributions of reported cases of *Escherichia coli* O157:H7 infection in Ontario. Epidemiol Infect.

[16] Samuel MC, Vugia DJ, Shallow S, Marcus R, Segler S, McGivern T (2004). Epidemiology of sporadic *Campylobacter* infection in the United States and declining trend in incidence, FoodNet 1996–1999. Clin Infect Dis.

[17] Guerin MT, Martin SW, Darlington GA (2005). Temporal clusters of *Salmonella* serovars in humans in Alberta, 1990–2001. Can J Public Health.

[18] Meldrum RJ, Griffiths JK, Smith RMM, Evans MR (2005). The seasonality of human *Campylobacter* infection and *Campylobacter* isolates from fresh, retail chicken in Wales. Epidemiol Infect.

[19] Tam CC, Rodriguez LC, O'Brien SJ, Hajat S (2006). Temperature dependence of reported *Campylobacter* infection in England, 1989–1999. Epidemiol Infect.

[20] Kovats RS, Edwards SJ, Hajat S, Armstrong BG, Ebi KL, Menne B (2004). The effect of temperature on food poisoning: a time-series analysis of salmonellosis in ten European countries. Epidemiol Infect.

[21] Sandberg M, Nygård K, Meldal H, Valle PS, Kruse H, Skjerve E (2006). Incidence trend and risk factors for *Campylobacter* infections in humans in Norway. BMC Public Health.

[22] Ekdahl K, Andersson Y (2004). Regional risks and seasonality in travel-associated campylobacteriosis. BMC Infect Dis.

[23] Green CG, Krause DO, Wylie JL (2006). Spatial analysis of *Campylobacter* infection in the Canadian province of Manitoba. Int J Health Geogr.

[24] Kistemann T, Zimmer S, Vågsholm I, Andersson Y (2004). GIS-supported investigation of human EHEC and cattle VTEC O157 infections in Sweden: geographical distribution, spatial variation and possible risk factors. Epidemiol Infect.

[25] Odoi A, Martin SW, Michel P, Middleton D, Holt J, Willson J (2004). Investigation of clusters of giardiasis using GIS and a spatial scan statistic. Int J Health Geogr.

[26] Statistics Canada. Census of Canada, 1996, GeoRef CD-ROM (cat. no. 92F0085XCB). Ottawa: Statistics Canada;1996.

[27] Long JS, Freese J. Regression models for categorical dependent variables using Stata. College Station: Stata Press; 2001.

[28] Cleveland RB, Cleveland WS, McRae JE, Terpenning I (1990). STL: a seasonal-trend decomposition procedure based on loess. J Off Stat.

[29] Kulldorff M, Feuer EJ, Miller BA, Freedman LS (1997). Breast cancer clusters in the northeast United States: a geographic analysis. Am J Epidemiol.

[30] Kulldorff M, Information Management Services Inc. SaTScan(tm) v8.0: Software for the spatial and space-time scan statistics. 2009. http://www.satscan.org. Accessed 30 Jan 2016.

[31] Corporation S (2007). Stata. 9.2 ed.

[32] R Development Core Team. R: a language and environment for statistical computing. 2.4.1 ed. Vienna: R Foundation for Statistical Computing; 2006. http://www.satscan.org. Accessed 30 Jan 2016.

[33] Spatial DMTI (2004). Unique enhanced and multiple enhanced postal codes.

[34] Inc ESRI (2005). ArcGIS desktop: release 10.

[35] Berke O. Choropleth mapping of regional count data of *Echinococcus multilocularis* among red foxes in lower Saxony, Germany. Prev Vet Med. 2001;52:119–31. doi:10.1016/S0167-5877(01)00246-X. 10.1016/s0167-5877(01)00246-x 11679170

[36] Bivand R, Anselin L, Berke O, Bernat A, Carvalho M, Chun Y (2008). SPDEP: spatial dependence: weighting, schemes, statistics and models. 0.4-20 ed.

[37] Berke O (2004). Exploratory disease mapping: kriging the spatial risk function from regional count data. Int J Health Geogr.

[38] Chen J, Roth RE, Naito AT, Lengerich EJ, Maceachren AM (2008). Geovisual analytics to enhance spatial scan statistic interpretation: an analysis of U.S. Cervical cancer mortality. Int J Health Geogr.

[39] Kulldorff M, Mostashari F, Duczmal L, Katherine Yih W, Kleinman K, Platt R (2007). Multivariate scan statistics for disease surveillance. Stat Med.

[40] Kulldorf M (2005). Scan Statistics for Geographical Disease Surveillance: An Overview. Spatial and Syndromic Surveillance for Public Health.

[41] Gregorio DI, DeChello LM, Samociuk H, Kulldorff M (2005). Lumping or splitting: seeking the preferred areal unit for health geography studies. Int J Health Geogr.

[42] Chhetri BK, Berke O, Pearl DL, Bienzle D (2014). Disparities in spatial prevalence of feline retroviruses due to data aggregation: a case of the modifiable areal unit problem. J Vet Med.

[43] Arsenault J, Michel P, Berke O, Ravel A, Gosselin P (2013). How to choose geographical units in ecological studies: proposal and application to campylobacteriosis. Spat Spatiotemporal Epidemiol.

[44] Department of the Environment (2009). Know your H2O. Domestic water quality monitoring.

[45] Valcour JE, Michel P, McEwen SA, Wilson JB (2002). Associations between indicators of livestock farming intensity and incidence of human Shiga toxin-producing *Escherichia coli* infection. Emerg Infect Dis.

[46] Environment Canada (2001). Threats to sources of drinking water and aquatic ecosystem health in Canada NWRI scientific assessment report series No. 1.

[47] Ong C, Moorehead W, Ross A, Isaac-Renton J (1996). Studies of *Giardia* spp. And *Cryptosporidium* spp. In two adjacent watersheds. Appl Environ Microbiol.

[48] New Brunswick Department of Agriculture. Agriculture, Fisheries and Aquaculture Sectors in Review 2002. Fredericton: Government of New Brunswick; 2002.

[49] Koning CW, Saffran KA, Little JL, Fent L. Water quality monitoring: the basis for watershed management in the Oldman River Basin, Canada. Water Sci Technol. 2006;53:153–61. doi:10.2166/wst.2006.308. 10.2166/wst.2006.308 16838699

[50] New Brunswick Forest Products Association. New Brunswick Forestry at a Glance. Fredericton: Government of New Brunswick; 2007.

[51] National Council for Air and Stream Improvement, Inc. (NCASI) (2009). Effects of forest management on water resources in Canada: a research review.

[52] Laupland KB, Church DL (2005). Population-based laboratory surveillance for *Giardia* sp. And *Cryptosporidium* sp. Infections in a large Canadian health region. BMC Infect Dis.

[53] Innocent GT, Mellord DJ, McEwen SA, Reilly WJ, Smallwood J, Locking ME (2005). Spatial and temporal epidemiology of sporadic human cases of *Escherichia coli* O157 in Scotland, 1996–1999. Epidemiol Infect.

[54] Miller G, Dunn GM, Smith-Palmer A, Ogden ID, Strachan NJC (2004). Human campylobacteriosis in Scotland: seasonality, regional trends and bursts of infection. Epidemiol Infect.

[55] Lal A, Hales S, French N, Baker MG (2012). Seasonality in human zoonotic enteric disease: a systematic review. PLoS One.

[56] Mac Kenzie WR, Hoxie NJ, Proctor ME, Gradus MS, Blair KA, Peterson DE (1994). A massive outbreak in Milwaukee of *Cryptosporidium* infection transmitted through the public water supply. N Engl J Med.

[57] Patz JA (2001). Public health risk assessment linked to climatic and ecological change. Hum Ecol Risk Assess: Int J.

[58] Rose JB, Epstein PR, Lipp EK, Sherman BH, Bernard SM, Patz JA (2001). Climate variability and change in the United States: potential impacts on water- and foodborne diseases caused by microbiologic agents. Environ Health Perspect.

[59] Schuster CJ, Ellis AG, Robertson WJ, Charron DF, Aramini JJ, Marshall BJ (2005). Infectious disease outbreaks related to drinking water in Canada, 1974–2001. Can J Public Health.

[60] Atherholt TB, LeChevallier MW, Norton WD, Rosen JS (1998). Effect of rainfall on *Giardia* and crypto. J Am Water Works Assoc.

[61] Hydat: Archived Hydrometic Data [database on the Internet]. Environment Canada. 2006. Accessed: 28 April 2004

[62] Phillips D (1990). The climates of Canada.

[63] van de Giessen AW, Bouwknegt M, Dam-Deisz WDC, van Pelt W, Wannet WJB, Visser G (2006). Surveillance of *Salmonella* spp. And *Campylobacter* spp. In poultry production flocks in the Netherlands. Epidemiol Infect.

[64] Wilson JB, Clarke RC, Renwick SA, Rahn K, Johnson RP, Karmali MA (1996). Vero cytotoxigenic *Escherichia coli* infection in dairy farm families. J Infect Dis.

[65] Statistics Canada (2001). Census of Canada, 2001, profile of Age and Sex, for Canada, provinces, territories, census divisions and census subdivisions.

[66] Government of New Brunswick (2014). New Brunswick communicable disease 2013 annual report.

